# Identification of novel proteins associated with intelligence by integrating genome-wide association data and human brain proteomics

**DOI:** 10.1371/journal.pone.0319278

**Published:** 2025-02-21

**Authors:** Zheng Zhang, Yousong Zhu, Junlong Zhang, Wenbin He, Cheng Han

**Affiliations:** 1 Shanxi Key Laboratory of Chinese Medicine Encephalopathy, Jinzhong, China; 2 National International Joint Research Center for Molecular Traditional Chinese Medicine, Jinzhong, China; 3 Basic Medical College of Shanxi University of Chinese Medicine, Jinzhong, China; Cyprus International University Faculty of Engineering: Uluslararasi Kibris Universitesi Muhendislik Fakultesi, TÜRKIYE

## Abstract

While genome-wide association studies (GWAS) have identified genetic variants associated with intelligence, their biological mechanisms remain largely unexplored. This study aimed to bridge this gap by integrating intelligence GWAS data with human brain proteomics and transcriptomics. We conducted proteome-wide (PWAS) and transcriptome-wide (TWAS) association studies, along with enrichment and protein-protein interaction (PPI) network analyses. PWAS identified 44 genes in the human brain proteome that influence intelligence through protein abundance regulation (FDR *P* <  0.05). Causal analysis revealed 36 genes, including GPX1, involved in the cis-regulation of protein abundance (*P* <  0.05). In independent PWAS analyses, 17 genes were validated, and 10 showed a positive correlation with intelligence (*P* <  0.05). TWAS revealed significant SNP-based heritability for mRNA in 28 proteins, and cis-regulation of mRNA levels for 20 genes was nominally associated with intelligence (FDR *P* <  0.05). This study identifies key genes that bridge genetic variants and protein-level mechanisms of intelligence, providing novel insights into its biological pathways and potential therapeutic targets.

## 1. Introduction

Intelligence refers to an individual’s ability to learn from experience, adapt, shape, and select environments, and is a frontier field in behavioral genetics research [1]. Intelligence has public health significance as it impacts academic performance, future personal health, and social well-being [2]. As a typical complex trait, intelligence is influenced by both genetic and environmental factors and exhibits high heritability. Intelligence is more predictive of important educational, occupational, and health outcomes than any other trait. In the 1970s and 1980s, debates over the genetic versus environmental influences on intelligence spurred larger and higher-quality family, twin, and adoption studies. These studies consistently demonstrated that genetics play a significant role in individual differences in intelligence.

Recent genome-wide association studies (GWAS) have successfully identified genetic sequence variations that account for 20% of the 50% heritability of intelligence [3]. Furthermore, a meta-analysis of GWAS in 269,867 individuals clarified the genetic associations with intelligence, identifying 205 associated genomic loci (190 of which were novel) and 1,016 related genes (939 of which were novel) [4]. These genes provide new insights for exploring the molecular mechanisms of intelligence.

Proteins are the most effective biomarkers and therapeutic targets [5,6] as they represent the primary functional components of cellular and biological processes and are the final products of gene expression [7]. Advances in mass spectrometry and spatial proteomics have enabled high-resolution mapping of protein networks in the human brain, providing a foundation for linking genetic variation to cognitive traits [8]. Previous studies have found that certain specific proteins are associated with intelligence or neurodegenerative diseases, such as NRX1A and periostin [9]. Recent research further indicates a significant association between proteins and intelligence traits [10]. Exploring proteins in greater depth can help us uncover the biological basis of intelligence and provide new avenues for enhancing cognitive function.

Transcriptome-wide association studies (TWAS) are a method used to investigate the correlation between the transcriptome and each genomic locus [11]. Similarly, proteome-wide association studies (PWAS) integrate GWAS data with proteomics data to identify candidate genes associated with a given trait [12].

In this study, we integrated intelligence GWAS data with human brain proteomics PWAS to identify risk genes associated with the proteome and transcriptome of intelligence.

## 2. Materials and methods

### 2.1. Data sources

#### 2.1.1. GWAS summary statistics.

We utilized the most extensive available intelligence meta-GWAS summary statistics, published by Savage et al. in 2018 [4]. The sample consists of 269,867 individuals from 14 independent epidemiological cohorts of European ancestry, including 9,295,118 genetic variation loci that passed quality testing.

#### 2.1.2. Brain proteomic and genetic data.

We used the discovery dataset from the Religious Order Study and Rush Memory and Aging Project (ROS/MAP) [13] and the Banner Sun Health Research Institute (Banner) [14] as the replication dataset. Protein data were obtained from human dorsolateral prefrontal cortex (dPFC) tissues, and matched genotyping was performed. Proteomic analysis utilized isobaric tandem mass tag peptide labeling followed by liquid chromatography-mass spectrometry.

Participants in the ROS/MAP cohort underwent genotyping using either whole-genome sequencing or genome-wide genotyping with platforms such as the Illumina OmniQuad Express or Affymetrix GeneChip 6.0. The detailed method can be described by Wingo et al [15]. After processing, the PWAS included 8,356 proteins from 376 individuals in the ROS/MAP dataset and 8,168 proteins from 152 individuals in the Banner dataset.

#### 2.1.3. Brain transcriptomic data.

The study analyzed brain transcriptome data from postmortem samples of 783 individuals of European descent, drawn from the ROS/MAP, Mount Sinai Brain Bank, and Mayo studies. The primary focus was on gene expression in the dorsolateral prefrontal cortex (dPFC), alongside other regions including the frontal cortex, temporal cortex, inferior frontal gyrus, superior temporal gyrus, and perirhinal gyrus. RNA-seq data underwent comprehensive quality control and normalization, as previously outlined [16]. Additionally, genome-wide genotyping was conducted for participants with transcriptomic data, a total of 13,650 genes from 888 reference brain transcriptomes were retained for the TWAS after quality control.

### 2.2. Statistical approach

#### 2.2.1. PWAS and TWAS.

We used the FUSION standard process to integrate brain protein/gene data with intelligence GWAS. Specifically, we first screened out proteins/genes with significant heritability based on heritability (*P* <  0.01). Five different predictive models (top1, blup, lasso, ennet, and bslmm) were then used to construct protein models, and the best model for each protein/gene was selected based on its predictive power. Next, the effect size Z value of intelligence GWAS was calculated, which represents the standardized score quantifying the deviation of the effect size of a given protein/gene from the mean effect size. This Z value was then weighted by the selected predictive model to estimate the protein/gene effect on intelligence. For PWAS results, we performed multiple tests using Bonferroni correction, and proteins with PWAS.*P* <  2.86 × 10^−5^ (0.05/1749) were considered significant. For TWAS results, false discovery rate (FDR) correction was used, and genes with *P* <  0.05 after correction were considered significantly correlated with intelligence.

#### 2.2.2. Causal analysis.

To determine causal relationships from our PWAS findings, we utilized two independent methods. For Bayesian colocalization analysis [17], we used the COLOC tool within the FUSION software to estimate the posterior probability that the same variant affects both GWAS and protein quantitative trait locus (pQTL) signals. Under this framework, five hypotheses (H0 to H4) were evaluated, with H4 suggesting a shared causal SNP. Causality was established if the posterior probability for H4 exceeded 0.5. To further validate these relationships, we applied the SMR method [18], using pQTL data and intelligence GWAS data. Significant causal associations were confirmed with an adjusted *P*-value <  0.05 for SMR and an unadjusted *P*-value >  0.05 for the HEIDI test.

#### 2.2.3. PPI and GO enrichment.

For the investigation of causal genes implicated in three diseases, we employed the STRING database to perform an extensive network analysis. In this visualization, the thickness of the line represents the strength of the interaction between two nodes, and we only reserved connections with an interaction score greater than 0.4, with different node colors representing different protein communities. Additionally, we conducted functional enrichment analysis for causal genes pertinent to three categories of diseases using the Metascape online platform [19]. We select the pathways with *P* <  0.05 (with FDR adjusted) as the significant result.

## 3. Result

### 3.1. Discovery PWAS of intelligence

We integrated human brain proteomics with the latest intelligence GWAS results, using the FUSION pipeline to perform a PWAS on intelligence. The human brain proteome was generated from the dorsolateral prefrontal cortex (dPFC) of 376 European ancestry participants from the ROS/MAP. After quality control, the proteome consisted of 8,356 proteins, of which 1,469 had significant single nucleotide polymorphism (SNP) heritability (*P* <  0.01) and were included in the PWAS. The intelligence GWAS summary statistics were sourced from the latest genome-wide association meta-analysis by Savage et al., which included 269,867 participants of European ancestry.

**Fig 1:**
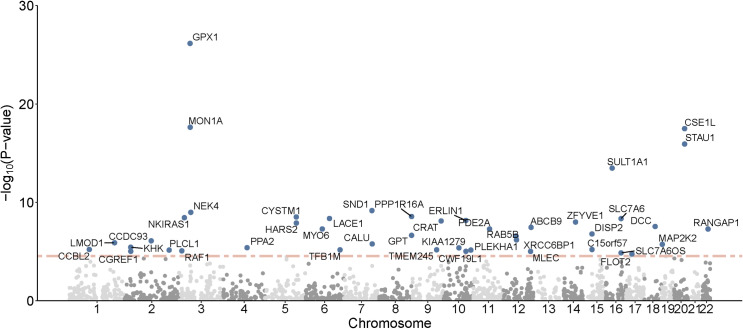
Manhattan plot of the discovery of intelligence-related PWAS. The intelligence GWAS (N =  269,867) is combined with the ROS/MAP proteome findings (N =  376). Each point represents a single association test between a gene and intelligence, ordered by the genomic position on the x-axis and the association strength on the y-axis, represented as −log10 (*P*) of the z-score test. A total of 44 cis-regulated brain proteins associated with intelligence were identified, with FDR *P* <  0.05. The red horizontal line indicates the FDR significance threshold of *P* <  0.05, set at the unadjusted maximum *P*-value below this threshold (*P* =  1.75 ×  10 ^−5^).

The PWAS identified 44 genes whose cis-regulated brain protein levels were associated with intelligence (FDR *P* <  0.05) (Fig 1 and Table 1). To further evaluate whether cis-regulated brain protein expression mediated the association between these 44 genes’ genetic variation and intelligence, we applied COLOC and SMR analyses to the same discovery dataset [18]. Multiple genes showed significant colocalization and causal associations (Supplementary Table S1 in S1 File). The COLOC analysis revealed that 29 genes, including GPX1, had an extremely high probability of colocalization. The SMR analysis indicated that 37 genes, including GPX1, had significant causal relationships (*P* <  0.05). We then performed heterogeneity testing using the HEIDI tool [18] to distinguish between pleiotropy/causal effects and linkage relationships for these 37 genes. HEIDI results indicated that 10 of the 37 genes may be significant due to linkage disequilibrium, while 27 were consistent with pleiotropy or causal relationships (Supplementary Table S1 in S1 File). SMR and HEIDI suggested that 36 genes, including GPX1, may be related to intelligence through cis-regulated brain protein abundance (Table 1). A total of 20 genes, including GPX1, exhibited high colocalization probabilities and causality, confirmed by both COLOC and SMR analyses.

**Table 1:** The discovery Intelligence PWAS identified 44 significant genes, of which 17 were found in the confirmation PWAS, and 14 replicated.

ID	CHR	START	END	Discovery		Replication	
				ROSMAP.Z	ROSMAP.P	BANNER.Z	BANNER.P
GPX1	3	49394609	49396033	-10.73	7.07 × 10^‒27^	‒10.79	3.97 × 10^‒27^
MON1A	3	49946302	49967606	8.74	2.33 × 10^‒18^	–	–
CSE1L	20	47662849	47713489	‒8.70	3.21 × 10^‒18^	‒6.48	9.29 × 10^‒11^
STAU1	20	47729878	47804904	‒8.29	1.18 × 10^‒16^	–	–
SULT1A1	16	28616903	28634946	7.59	3.30 × 10^‒14^	8.32	8.61 × 10^‒17^
SND1	7	127292234	127732661	‒6.17	6.85 × 10^‒10^	–	–
NEK4	3	52744800	52804965	‒6.10	1.03 × 10^‒9^	–	–
PPP1R16A	8	145703352	145727504	5.95	2.73 × 10^‒9^	–	–
CYSTM1	5	139554227	139661637	5.93	3.05 × 10^‒9^	5.93	3.05 × 10^‒9^
NKIRAS1	3	23933151	23988082	‒5.90	3.57 × 10^‒9^	–	–
LACE1	6	108616098	108847999	5.87	4.32 × 10^‒9^	–	–
SLC7A6	16	68298433	68335722	‒5.87	4.45 × 10^‒9^	–	–
ERLIN1	10	101909851	101948091	‒5.79	6.87 × 10^‒9^	‒5.03	4.85 × 10^‒7^
CRAT	9	131857089	131873468	‒5.77	7.79 × 10^‒9^	0.57	0.571
ZFYVE1	14	73436159	73493920	5.73	1.00 × 10^‒8^	–	–
HARS2	5	140071011	140078889	‒5.69	1.25 × 10^‒8^	–	–
DCC	18	49866542	51057784	‒5.55	2.82 × 10^‒8^	–	–
ABCB9	12	123405498	123466196	5.52	3.45 × 10^‒8^	–	–
MYO6	6	76458909	76629254	‒5.45	5.02 × 10^‒8^	‒5.49	3.93 × 10^‒8^
PDE2A	11	72287185	72385635	5.45	5.04 × 10^‒8^	–	–
RANGAP1	22	41641615	41682255	‒5.45	5.10 × 10^‒8^	–	–
DISP2	15	40650436	40663257	‒5.24	1.61 × 10^‒7^	–	–
GPT	8	145728356	145732557	5.18	2.23 × 10^‒7^	5.28	1.33 × 10^‒7^
RAB5B	12	56367697	56388490	‒5.14	2.70 × 10^‒7^	–	–
XRCC6 BP1	12	58335324	58351052	4.98	6.49 × 10^‒7^	–	–
CCDC93	2	118673054	118771709	4.93	8.06 × 10^‒7^	–	–
LMOD1	1	201865580	201915715	4.85	1.26 × 10^‒6^	4.00	6.28 × 10^‒5^
CALU	7	128379346	128411861	4.79	1.67 × 10^‒6^	4.63	3.63 × 10^‒6^
MAP2K2	19	4090319	4124126	4.77	1.86 × 10^‒6^	1.88	0.06
KHK	2	27309615	27323640	‒4.63	3.59 × 10^‒6^	‒4.21	2.57 × 10^‒5^
PPA2	4	106290234	106395238	4.61	4.02 × 10^‒6^	3.16	0.002
KIAA1279	10	70748487	70776738	‒4.60	4.18 × 10^‒6^	–	–
CCBL2	1	89401456	89458636	4.52	6.06 × 10^‒6^	4.47	7.93 × 10^‒6^
C15orf57	15	40820882	40857256	4.52	6.21 × 10^‒6^	–	–
TMEM245	9	111777432	111882225	‒4.51	6.56 × 10^‒6^	‒0.65	0.513
TFB1M	6	155578643	155635627	‒4.51	6.64 × 10^‒6^	–	–
PLEKHA1	10	124134212	124191867	‒4.49	6.99 × 10^‒6^	3.49	4.86 × 10^‒4^
PLCL1	2	198669426	199437305	4.49	7.04 × 10^‒6^	–	–
RAF1	3	12625100	12705725	4.45	8.43 × 10^‒6^	–	–
CWF19L1	10	101992055	102027437	4.43	9.38 × 10^‒6^	–	–
CGREF1	2	27321757	27341995	4.43	9.39 × 10^‒6^	–	–
MLEC	12	121124672	121139667	4.43	9.63 × 10^‒6^	–	–
SLC7A6OS	16	68318406	68344849	‒4.35	1.36 × 10^‒5^	–	–
FLOT2	17	27206353	27224697	‒4.30	1.74 × 10^‒5^	‒4.31	1.61 × 10^‒5^

### 3.2. Replication PWAS of intelligence

To increase the credibility of our findings, we performed a replication PWAS for intelligence using proteomic and GWAS results that were not included in our discovery analysis. The replication human brain proteome was generated from the dPFC of 152 European-ancestry participants recruited by the Banner Sun Health Research Institute. After quality control, the proteome consisted of 8,168 proteins, of which 1,139 proteins had significant SNP-based heritability (*P* <  0.01) and were included in the replication PWAS. Seventeen genes were replicated in the independent PWAS for intelligence, providing greater confidence in our results (Fig 1 and Table 1). Of these, 10 genes were positively correlated and 7 were negatively correlated. Twenty-seven of the 44 significant proteins identified in the discovery PWAS were not detected in the replication PWAS. CRAT, MAP2K2, and TMEM245 were analyzed, but the results in the replication cohort were not significant (*P* >  0.05) (Table 1).

### 3.3. Examination of the potential intelligence-related proteins at the mRNA level

The brain transcriptome data for this study were primarily derived from postmortem brain samples of 783 European ancestry participants from the ROS/MAP, Mayo, and Mount Sinai Brain Bank studies, focusing on the frontal cortex. Among the 13,650 mRNAs that passed quality control, 6,735 exhibited significant SNP-based heritability and were included in the TWAS. The intelligence TWAS using the FUSION pipeline identified 20 genes whose cis-regulated brain mRNA expression was associated with intelligence (FDR *P* <  0.05) (Supplementary Table S2 in S1 File). All 44 proteins identified in the discovery PWAS were analyzed at the mRNA level; however, only 28 of them, including GPX1, exhibited significant SNP-based mRNA heritability estimates (Supplementary Table S2 in S1 File). The TWAS revealed that 20 of these 28 genes had nominally significant associations with intelligence at the cis-regulated mRNA level, with 10 of these genes showing consistent directionality of effects on both mRNA and protein levels.

Additionally, among the 44 intelligence-related genes, 16 genes showed no evidence of association with intelligence at the mRNA level in TWAS, including those that were not heritable and thus not included in the analysis. Interestingly, 6 of these 16 genes had significant findings in the discovery PWAS and were replicated (GPT, MAP2K2, KHK, CCBL2, PLEKHA1, and FLOT2; Table 1). This suggests that PWAS provides novel insights into the pathophysiological mechanisms of intelligence beyond what TWAS has revealed.

### 3.4. Enrichment Analysis of Pathways Based on Intelligence-Causal Genes

**Fig 2:**
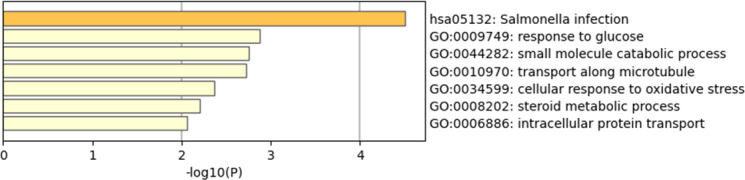
Enrichment analysis of causal gene pathways related to intelligence.

To further identify the functions of the candidate proteins, we performed enrichment analysis using the coding genes of the proteins identified by PWAS. The result of enrichment revealed that intelligence-causative genes are significantly involved in various biological processes, including Salmonella infection, glucose response, small molecule metabolic processes, microtubule transport, cellular responses to oxidative stress, steroid metabolism, and intracellular protein transport (Fig 2). These findings were derived from proteomic data, providing insights into the functional roles of these proteins in intelligence-related pathways.

### 3.5. Protein-Protein Interaction Networks in Intelligence

**Fig 3:**
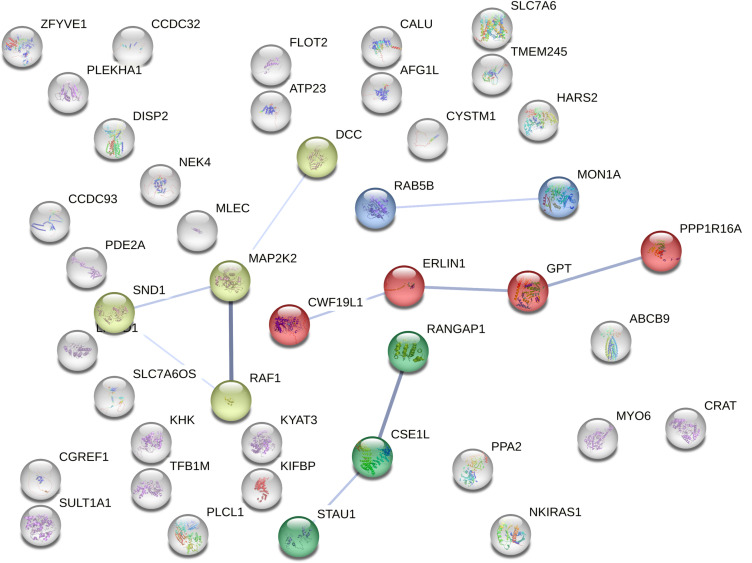
PPI network and pathways of the 44 significant proteins associated with intelligence in the PWAS. The lines represent physical PPIs, with the thickness of the lines proportional to the strength of the PPI evidence. Enrichment of pathways was determined using the hypergeometric test with Bonferroni correction for multiple tests.

We investigated the connectivity among the 44 intelligence-related proteins identified in the PWAS using the STRING database and discovered a protein community based on protein-protein interactions (PPIs). A module is defined as a group of proteins that have tighter connections with each other than with other protein groups. Community 1 includes RANGAP1, CSE1L, and STAU1; Community 2 includes SND1, MAP2K2, RAF1, and DCC; Community 3 includes CWF19L1, ERLIN1, GPT, and PPP1R16A; and Community 4 includes MON1A and RAB5B (Fig 3).

## 4. Discussion

Intelligence is a typical complex trait influenced by both genetic and environmental factors, exhibiting high heritability. It is more representative than any other characteristic in predicting significant educational, occupational, and health outcomes. For instance, there is ample evidence that intelligence has an independent causal relationship with the risk of Alzheimer’s disease (AD), attention deficit hyperactivity disorder (ADHD), and schizophrenia [4,20]. Identifying genetic targets that influence intelligence is a critical objective in human genetics research, particularly significant for enhancing the understanding and development of cognitive abilities.

Although previous studies have identified the functional relevance of tissue proteins and the development of brain function, the potential biological mechanisms between tissue proteins and intelligence remain to be elucidated [21]. In this study, we employed a range of analytical techniques to investigate the functional associations between protein biomarkers in the brain and intelligence. We identified 44 candidate genes associated with changes in brain protein abundance related to intelligence. Among these, 17 genes were replicated in independent PWAS analyses of intelligence, providing higher confidence in our findings. Additionally, we discovered that GPX1 and 19 other genes exhibited co-localization and causal inference related to intelligence in the brain PWAS, while the associations of genes such as CSE1L with intelligence were supported at the brain transcript level. Enrichment analyses revealed that these genes participate in various biological processes, including responses to Salmonella infection, glucose metabolism, small molecule metabolic processes, microtubule transport, cellular responses to oxidative stress, steroid metabolism, and intracellular protein transport. These results suggest that these genes may collectively influence intelligence performance by regulating these critical pathways. Further analysis indicates that these genes may synergistically participate in the regulation of the target traits at the transcriptomic and proteomic levels, highlighting their potential roles in related biological mechanisms. This finding provides robust support and promising directions for subsequent mechanistic studies and the development of therapeutic targets.

Our analysis involves genes previously studied in the context of intelligence. Prior research has identified GPT, an enzyme involved in brain amino acid metabolism, as a candidate gene for intelligence [22]. Its function may be related to cognitive abilities and plays a crucial role in the complex behaviors of neurons. Additionally, studies have shown that the antioxidant enzyme GPX1 is widely expressed in brain tissue and is significantly associated with cognitive function [23]. Moreover, dietary and exercise interventions can enhance cognitive function by regulating GPX levels [24–26], which aligns closely with our findings.

Furthermore, CSE1L is associated with apoptosis and proliferation, demonstrating a strong correlation with intelligence performance in GWAS [27]. Previous studies have indicated that patients with mutations in MAP2K2 may exhibit better functional preservation in intelligence [28]. Specifically, in terms of neurodevelopmental functions, patients with mutations in the MAP2K2 gene show a lower incidence of intellectual disability (ID) compared to those with mutations in other genes, such as BRAF and MAP2K1, with an incidence rate of only 25%.

Additionally, previous studies have identified NEK4, ERLIN1, PLCL1, SULT1A1, CYSTM1, and PLEKHA1 as candidate genes for intelligence, which aligns with our findings. Specifically, NEK4, one of the largest members of the NEK family, is involved in the DNA damage response. Consistent evidence suggests its association with schizophrenia and bipolar disorder [29]. As a critical gene in cell cycle regulation, NEK4 may play a key role in neuronal proliferation and survival, thereby influencing intelligence performance.

Furthermore, research has shown that PLCL1 is significantly associated with green exposure and is involved in neurotransmitter clearance, affecting the development of intelligence in children [30]. Additionally, PLCL1 has been linked to hereditary dyslexia and ADHD [31], suggesting potential implications during the process of intelligence development.

While SULT1A1 may have some association with intelligence, its function in the brain has not been thoroughly investigated, and further functional studies are needed to validate its specific role [32]. CYSTM1 is a candidate gene that influences pregnancy and has been associated with body mass index and intelligence, indicating its significant role in developmental regulation [33]. Additionally, PLEKHA1 is related to intelligence through its involvement in protein synthesis, energy metabolism, and amino acid metabolism [34].

This study offers industrial feasibility in areas such as drug development, biomarker identification, and precision medicine by providing insights into proteins and genes associated with intelligence, which could inform therapeutic and diagnostic advancements for cognitive disorders.

In conclusion, this study provides significant contributions to the understanding of the genetic and proteomic foundations of intelligence. We conducted the largest and most comprehensive pQTL analysis of intelligence PWAS to date, utilizing the latest summary statistics from GWAS. By replicating the PWAS with an independent human brain proteome and validating causal relationships through MR analyses, we strengthened the confidence in the identified risk proteins. The integration of PWAS and TWAS analyses allowed us to explore the complex relationships between mRNA and protein levels associated with intelligence while identifying four core protein modules CWF19L1, ERLIN1, GPT, and PPP1R16A through PPIs, shedding light on critical biological pathways that influence cognitive functions.

However, the current study has several limitations. First, while pQTL and eQTL mapping provide valuable insights, they cannot fully capture all GWAS signals or comprehensively interpret the functional roles of genes in the biological pathways underlying intelligence. A single-layer analysis, such as at the protein level, may overlook critical interactions across molecular layers. Future studies incorporating multi-omics approaches, such as methylation quantitative trait loci (mQTL), single-cell sequencing, and whole-genome sequencing, are essential to uncover the complete molecular mechanisms associated with intelligence and to inform the development of tailored therapeutic strategies [35,36]. Second, the limited sample size and racial specificity of the proteomic dataset may constrain the generalizability of the findings. Expanding the scale and diversity of brain proteomic data across different populations and age groups will be crucial for improving the robustness of the results, enabling more precise effect estimates, and ensuring broader applicability. Additionally, addressing potential technical biases introduced by varying genotyping platforms used across datasets could further enhance the reliability of the conclusions.

## Supporting information

S1 File Table S1. Colocalization and causal analysis results for intelligence genes. **Table S2.** TWAS Results for Intelligence.
(DOCX)
