# Supplementary material for: Identification of novel proteins associated with intelligence by integrating genome-wide association data and human brain proteomics
Source: PLoS One. 2025 Feb 21;20(2):e0319278. doi: 10.1371/journal.pone.0319278 (PMC11844858; doi:10.1371/journal.pone.0319278)
Supplement: S1 File — Colocalization and causal analysis results for intelligence genes. Table S2. TWAS Results for Intelligence. (DOCX) [file pone.0319278.s001.docx]

**Table S1. Colocalization and causal analysis results for intelligence genes**

| ID | CHR | P0 | P1 | COLOC | | SMR | | | causal |
| --- | --- | --- | --- | --- | --- | --- | --- | --- | --- |
|  |  |  |  | COLOC.PP4 | COLOC.causal | SMR.P | HEIDI.P | SMR.causal |  |
| GPX1 | 3 | 49394609 | 49396033 | 0.992 | YES | 4.46×10^-16^ | 0.181 | YES | YES |
| MON1A | 3 | 49946302 | 49967606 | 0.695 | YES | - | - | NO | YES |
| CSE1L | 20 | 47662849 | 47713489 | 0.949 | YES | 4.29×10^-5^ | 0.179 | YES | YES |
| STAU1 | 20 | 47729878 | 47804904 | 0.855 | YES | 4.74×10^-4^ | 0.010 | NO | YES |
| SULT1A1 | 16 | 28616903 | 28634946 | 0.954 | YES | 1.33×10^-8^ | 0.047 | NO | YES |
| SND1 | 7 | 127292234 | 127732661 | 0.858 | YES | 0.354 | 0.100 | NO | YES |
| NEK4 | 3 | 52744800 | 52804965 | 0.779 | YES | 7.26×10^-5^ | 0.287 | YES | YES |
| PPP1R16A | 8 | 145703352 | 145727504 | 0.815 | YES | 0.003 | 0.847 | YES | YES |
| CYSTM1 | 5 | 139554227 | 139661637 | 0.42 | NO | 5.19×10^-6^ | 0.077 | YES | YES |
| NKIRAS1 | 3 | 23933151 | 23988082 | 0.081 | NO | 0.019 | 0.031 | NO | NO |
| LACE1 | 6 | 108616098 | 108847999 | 0.225 | NO | 0.006 | 0.873 | YES | YES |
| SLC7A6 | 16 | 68298433 | 68335722 | 0.234 | NO | - | - | NO | NO |
| ERLIN1 | 10 | 101909851 | 101948091 | 0.999 | YES | 6.18×10^-5^ | 0.004 | NO | YES |
| CRAT | 9 | 131857089 | 131873468 | 0.737 | YES | 0.003 | 0.727 | YES | YES |
| ZFYVE1 | 14 | 73436159 | 73493920 | 0.985 | YES | 0.002 | 0.954 | YES | YES |
| HARS2 | 5 | 140071011 | 140078889 | 0.768 | YES | 0.002 | 0.471 | YES | YES |
| DCC | 18 | 49866542 | 51057784 | 0.98 | YES | 4.12×10^-7^ | 0.276 | YES | YES |
| ABCB9 | 12 | 123405498 | 123466196 | 0.972 | YES | 0.256 | 0.047 | NO | YES |
| MYO6 | 6 | 76458909 | 76629254 | 0.993 | YES | 9.13×10^-7^ | 0.130 | YES | YES |
| PDE2A | 11 | 72287185 | 72385635 | 0.024 | NO | 0.010 | 0.955 | YES | YES |
| RANGAP1 | 22 | 41641615 | 41682255 | 0.204 | NO | 0.018 | 0.307 | YES | YES |
| DISP2 | 15 | 40650436 | 40663257 | 0.982 | YES | 8.27×10^-5^ | 0.373 | YES | YES |
| GPT | 8 | 145728356 | 145732557 | 0.814 | YES | 7.60×10^-4^ | 0.414 | YES | YES |
| RAB5B | 12 | 56367697 | 56388490 | 0.75 | YES | 0.008 | 0.312 | YES | YES |
| XRCC6BP1 | 12 | 58335324 | 58351052 | 0.027 | NO | 0.001 | 0.259 | YES | YES |
| CCDC93 | 2 | 118673054 | 118771709 | 0.981 | YES | 0.003 | 0.931 | YES | YES |
| LMOD1 | 1 | 201865580 | 201915715 | 0.896 | YES | 0.002 | 0.039 | NO | YES |
| CALU | 7 | 128379346 | 128411861 | 0.486 | NO | 9.01×10^-5^ | 0.008 | NO | NO |
| MAP2K2 | 19 | 4090319 | 4124126 | 0.304 | NO | 0.016 | - | NO | NO |
| KHK | 2 | 27309615 | 27323640 | 0.981 | YES | 1.06×10^-5^ | 0.200 | YES | YES |
| PPA2 | 4 | 106290234 | 106395238 | 0.04 | NO | 0.022 | 0.005 | NO | NO |
| KIAA1279 | 10 | 70748487 | 70776738 | 0.968 | YES | 1.62×10^-4^ | 0.006 | NO | YES |
| CCBL2 | 1 | 89401456 | 89458636 | 0.822 | YES | 4.92×10^-5^ | 0.058 | YES | YES |
| C15orf57 | 15 | 40820882 | 40857256 | 0.941 | YES | 0.013 | 0.463 | YES | YES |
| TMEM245 | 9 | 111777432 | 111882225 | 0.997 | YES | - | - | NO | YES |
| TFB1M | 6 | 155578643 | 155635627 | 0.806 | YES | 0.001 | 0.231 | YES | YES |
| PLEKHA1 | 10 | 124134212 | 124191867 | 0.708 | YES | 0.002 | 0.062 | YES | YES |
| PLCL1 | 2 | 198669426 | 199437305 | 0.335 | NO | 5.54×10^-4^ | 0.356 | YES | YES |
| RAF1 | 3 | 12625100 | 12705725 | 0.768 | YES | 0.024 | 0.251 | YES | YES |
| CWF19L1 | 10 | 101992055 | 102027437 | 0.281 | NO | 9.06×10^-5^ | 0.003 | NO | NO |
| CGREF1 | 2 | 27321757 | 27341995 | 0.312 | NO | 0.005 | 0.737 | YES | YES |
| MLEC | 12 | 121124672 | 121139667 | 0.223 | NO | 0.065 | 0.256 | NO | NO |
| SLC7A6OS | 16 | 68318406 | 68344849 | 0.074 | NO | 0.361 | 0.007 | NO | NO |
| FLOT2 | 17 | 27206353 | 27224697 | 0.856 | YES | 2.84×10^-4^ | 0.513 | YES | YES |

**Table S2. TWAS Results for Intelligence**

| ID | CHR | P0 | P1 | PWAS.Z | PWAS.P | TWAS.Z | TWAS.P |
| --- | --- | --- | --- | --- | --- | --- | --- |
| GPX1 | 3 | 49394609 | 49396033 | -10.73 | 7.07×10^-27^ | -8.81 | 1.20×10^-18^ |
| MON1A | 3 | 49946302 | 49967606 | 8.74 | 2.33×10^-18^ | - | - |
| CSE1L | 20 | 47662849 | 47713489 | -8.70 | 3.21×10^-18^ | -8.02 | 1.04×10^-15^ |
| STAU1 | 20 | 47729878 | 47804904 | -8.29 | 1.18×10^-16^ | -6.25 | 4.23×10^-10^ |
| SULT1A1 | 16 | 28616903 | 28634946 | 7.59 | 3.30×10^-14^ | 5.81 | 6.21×10^-9^ |
| SND1 | 7 | 127292234 | 127732661 | -6.17 | 6.85×10^-10^ | -6.62 | 3.64×10^-11^ |
| NEK4 | 3 | 52744800 | 52804965 | -6.10 | 1.03×10^-9^ | -5.03 | 5.01×10^-7^ |
| PPP1R16A | 8 | 145703352 | 145727504 | 5.95 | 2.73×10^-9^ | - | - |
| CYSTM1 | 5 | 139554227 | 139661637 | 5.93 | 3.05×10^-9^ | 4.13 | 3.64×10^-5^ |
| NKIRAS1 | 3 | 23933151 | 23988082 | -5.90 | 3.57×10^-9^ | -5.62 | 1.91×10^-8^ |
| LACE1 | 6 | 108616098 | 108847999 | 5.87 | 4.32×10^-9^ | -1.33 | 0.184 |
| SLC7A6 | 16 | 68298433 | 68335722 | -5.87 | 4.45×10^-9^ | - | - |
| ERLIN1 | 10 | 101909851 | 101948091 | -5.79 | 6.87×10^-9^ | -0.60 | 0.546 |
| CRAT | 9 | 131857089 | 131873468 | -5.77 | 7.79×10^-9^ | -4.28 | 1.84×10^-5^ |
| ZFYVE1 | 14 | 73436159 | 73493920 | 5.73 | 1.00×10^-8^ | -0.18 | 0.854 |
| HARS2 | 5 | 140071011 | 140078889 | -5.69 | 1.25×10^-8^ | - | - |
| DCC | 18 | 49866542 | 51057784 | -5.55 | 2.82×10^-8^ | - | - |
| ABCB9 | 12 | 123405498 | 123466196 | 5.52 | 3.45×10^-8^ | - | - |
| MYO6 | 6 | 76458909 | 76629254 | -5.45 | 5.02×10^-8^ | -3.74 | 1.85×10^-4^ |
| PDE2A | 11 | 72287185 | 72385635 | 5.45 | 5.04×10^-8^ | -1.83 | 0.067 |
| RANGAP1 | 22 | 41641615 | 41682255 | -5.45 | 5.10×10^-8^ | - | - |
| DISP2 | 15 | 40650436 | 40663257 | -5.24 | 1.61×10^-7^ | -3.48 | 4.95×10^-4^ |
| GPT | 8 | 145728356 | 145732557 | 5.18 | 2.23×10^-7^ | - | - |
| RAB5B | 12 | 56367697 | 56388490 | -5.14 | 2.70×10^-7^ | 0.38 | 0.702 |
| XRCC6BP1 | 12 | 58335324 | 58351052 | 4.98 | 6.49×10^-7^ | - | - |
| CCDC93 | 2 | 118673054 | 118771709 | 4.93 | 8.06×10^-7^ | -2.75 | 0.006 |
| LMOD1 | 1 | 201865580 | 201915715 | 4.85 | 1.26×10^-6^ | 1.23 | 0.22 |
| CALU | 7 | 128379346 | 128411861 | 4.79 | 1.67×10^-6^ | -0.57 | 0.569 |
| MAP2K2 | 19 | 4090319 | 4124126 | 4.77 | 1.86×10^-6^ | - | - |
| KHK | 2 | 27309615 | 27323640 | -4.63 | 3.59×10^-6^ | - | - |
| PPA2 | 4 | 106290234 | 106395238 | 4.61 | 4.02×10^-6^ | -3.07 | 0.002 |
| KIAA1279 | 10 | 70748487 | 70776738 | -4.60 | 4.18×10^-6^ | - | - |
| CCBL2 | 1 | 89401456 | 89458636 | 4.52 | 6.06×10^-6^ | - | - |
| C15orf57 | 15 | 40820882 | 40857256 | 4.52 | 6.21×10^-6^ | 3.90 | 9.53×10^-5^ |
| TMEM245 | 9 | 111777432 | 111882225 | -4.51 | 6.56×10^-6^ | -2.72 | 0.007 |
| TFB1M | 6 | 155578643 | 155635627 | -4.51 | 6.64×10^-6^ | -5.51 | 3.66×10^-8^ |
| PLEKHA1 | 10 | 124134212 | 124191867 | -4.49 | 6.99×10^-6^ | - | - |
| PLCL1 | 2 | 198669426 | 199437305 | 4.49 | 7.04×10^-6^ | 1.06 | 0.288 |
| RAF1 | 3 | 12625100 | 12705725 | 4.45 | 8.43×10^-6^ | - | - |
| CWF19L1 | 10 | 101992055 | 102027437 | 4.43 | 9.38×10^-6^ | 4.37 | 1.22×10^-5^ |
| CGREF1 | 2 | 27321757 | 27341995 | 4.43 | 9.39×10^-6^ | 2.96 | 0.003 |
| MLEC | 12 | 121124672 | 121139667 | 4.43 | 9.63×10^-6^ | 3.89 | 9.98×10^-5^ |
| SLC7A6OS | 16 | 68318406 | 68344849 | -4.35 | 1.36×10^-5^ | 2.48 | 0.0132 |
| FLOT2 | 17 | 27206353 | 27224697 | -4.30 | 1.74×10^-5^ | - | - |
